# 404 Mechanisms of a Dynamic Stability Protocol for Persons with Thumb Osteoarthritis – CORRIGENDUM

**DOI:** 10.1017/cts.2024.528

**Published:** 2024-05-07

**Authors:** Corey McGee, Halil Ibrahim Ergen, Paula Ludewig, Ann Brearley, Ann Van Heest, Erin Krebs

The above abstract published with an error in the author list. Halil Ibrahim Ergen was not included. The correct list can be seen below.

Corey McGee, Halil Ibrahim Ergen, Paula Ludewig, Ann Brearley, Ann Van Heest and Erin Krebs.

The original abstract has been corrected online to rectify these errors.
